# Expanding the clinical tumor phenotype of the *EPAS1*-asssociated tumor syndrome

**DOI:** 10.1210/clinem/dgag003

**Published:** 2026-01-10

**Authors:** Yasemin Cole, Sophie Howarth, Asna Javaid, Patrick Tarpey, Daniel Scoffings, Eamonn R Maher, Anna L Godfrey, Karel Pacak, Zhengping Zhuang, Hussam Alkaissi, Ruth T Casey

**Affiliations:** Department of Genomic Medicine, University of Cambridge, Cambridge CB2 0QQ, UK; Department of Endocrinology, Cambridge University Hospital, Cambridge Biomedical Research Centre, Addenbrooke’s Hospital, Cambridge CB2 0QQ, UK; Molecular Genetic Laboratories, Cambridge University Hospital, Cambridge Biomedical Research Centre, Addenbrooke’s Hospital, Cambridge CB2 0QQ, UK; Molecular Genetic Laboratories, Cambridge University Hospital, Cambridge Biomedical Research Centre, Addenbrooke’s Hospital, Cambridge CB2 0QQ, UK; Department of Radiology, Cambridge University Hospital, Cambridge Biomedical Research Centre, Addenbrooke’s Hospital, Cambridge CB2 0QQ, UK; Department of Genomic Medicine, University of Cambridge, Cambridge CB2 0QQ, UK; Aston Medical School, College of Health and Life Sciences, Birmingham B4 7ET, UK; Department of Haematology, Cambridge University Hospitals, NHS Foundation Trust, Cambridge CB2 0QQ, UK; Section on Medical Neuroendocrinology, Eunice Kennedy Shriver National Institute of Child Health and Human Development, National Institutes of Health (NIH), Bethesda, MD 20892, USA; Center for Adrenal Endocrine Tumors, AKESO, Prague 5 158 00, Czech Republic; Multidisciplinary Consortium for Adrenal Diseases, Faculty of Medicine and Faculty Hospital, Palacky University, Olomouc 775 15, Czech Republic; Neuro-Oncology Branch, National Cancer Institute, National Institutes of Health, Bethesda, MD 20892, USA; National Institute of Diabetes and Digestive and Kidney Diseases, NIH, Bethesda, MD 20892, USA; Department of Genomic Medicine, University of Cambridge, Cambridge CB2 0QQ, UK; Department of Endocrinology, Cambridge University Hospital, Cambridge Biomedical Research Centre, Addenbrooke’s Hospital, Cambridge CB2 0QQ, UK

**Keywords:** *EPAS1*, pancreatic neuroendocrine tumor, paraganglioma/pheochromocytoma, genotype-phenotype

## Abstract

**Context:**

Since the original discovery of the Pacak-Zhuang syndrome (PZS) in 2012, defined by the clinical triad of pheochromocytoma/paraganglioma (PPGL) and/or duodenal ampullar somatostatinoma with erythrocytosis, multiple multisystemic phenotypes have been identified in patients with somatic mosaic pathogenic variants in *EPAS1*/*HIF2A*. Deep phenotyping of patients along with evaluation of a transgenic murine model has led to the understanding of the role of HIF-2α in developmental processes, including tumor development. Interestingly, pancreatic neuroendocrine tumors (NETs) occur in von Hippel-Lindau disease and the *VHL* gene product regulates HIF-2α expression.

**Objective:**

Characterize pancreatic NETs in PZS.

**Methods:**

We have reviewed the clinical records in the index patient at Addenbrooke’s Hospital (UK) and a cohort of patients with PZS from the NIH were assessed for pancreatic NETs.

**Results:**

Herein, we describe a novel series from two institutions of patients with *EPAS1*-associated pancreatic neuroendocrine tumors including a case of a nonfunctioning pancreatic NET in association with an *EPAS1* somatic mosaic variant.

**Conclusion:**

This case study extends our current understanding of the phenotypic spectrum in PZS and links pancreatic NETs to an additional hypoxia-associated gene, namely *EPAS1*.

In the early 2000s, members of a family with erythrocytosis were found to have a novel germline *EPAS1/HIF2A* gain-of-function variant (1). At the same time, patients with von Hippel-Lindau (VHL)-associated Chuvash erythrocytosis (homozygous *VHL* c.598C > T variants) uniquely present with blood cell dyscrasias and elevated vascular endothelial growth factor in the absence of tumors (2). Taken together, this led to the concept that HIF-2α (encoded by *EPAS1/HIF2A*) may be involved in erythrocytosis. Mosaic postzygotic gain-of-function variants in *EPAS1/HIF2A* were then identified in 2 individuals with congenital erythrocytosis, pheochromocytoma/paraganglioma (PPGL), and duodenal ampullar somatostatinoma, connecting for the first time *EPAS1* to tumor development (3, 4).

Concurrent to the discovery of this syndrome, a transgenic murine model bearing a mosaic variant, introduced early in embryonic development, solidified our understanding of the role of *EPAS1* in PPGL development (5). This model reproduced the clinical spectrum of Pacak-Zhuang syndrome (PZS), including erythrocytosis and somatostatinoma-positive cells within the duodenal ampulla. Deep phenotyping and assessment of patients with PZS with the PPGL clinical program at the National Institutes of Health (NIH) (6, 7) have revealed that patients and mice have unexpected multisystemic malformations, likely a reflection of the spatiotemporal expression of HIF-2α during embryonic development (8). Beyond erythrocytosis and neural crest–derived PPGL tumors, *EPAS1* gain-of-function variants have been linked to ocular defects (eg, tortuous retinal vessels, optic nerve gliosis, morning glory anomalies, retinal and macular edema), systemic venule malformations (eg, cavernous malformations in the spinal vascular network), enlarged venous drainage of the central nervous system (Galen vein and dural sinuses), and neural tube defects (eg, neuraxial dysraphism) within patients and the corresponding transgenic mouse model (Fig. 1) (9-11).

**Figure 1:**
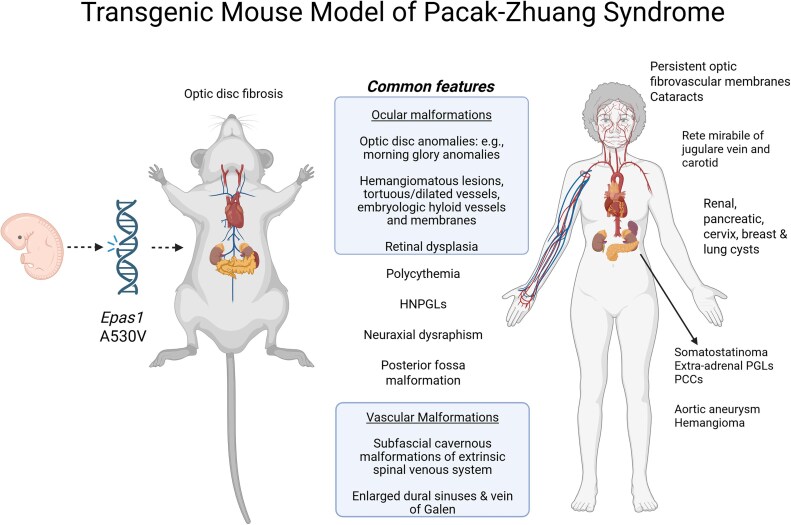
Summary of *EPAS1*-associated phenotypes in Pacak-Zhuang syndrome (PZS) and a transgenic murine model. Disease entities were compiled from previously reported publications (6, 9-11, 38). Phenotypes identified thus far in the *Epas1* murine model and in PZS patients are listed on the left- and right-hand sides, respectively, while the middle section lists common phenotypes. Created in BioRender. Cole, Y. (2025) https://biorender.com/g1a8rco.

Pancreatic neuroendocrine tumors (pNETs) are a feature of some inherited cancer syndromes, including multiple endocrine neoplasia type 1 (MEN1), von Hippel-Lindau (VHL) disease and, occasionally, in patients with germline pathogenic variants in the succinate dehydrogenase subunit B and subunit D gene (*SDHB* and *SDHD*, respectively) and neurofibromatosis (*NF1*) (12, 13). Extensive whole-exome and genome sequencing studies have revealed that a sizeable percentage of pNETs are heritable (17%) and can harbor germline and somatic pathogenic variants in *VHL*, *NF1*, and *ATRX*, similar to PPGLs, and genetic defects affecting the PI3K/mTOR pathway (eg, *TSC1/2*, *PTEN*) (14-16). Scarpa et al (14) identified a distinct cluster of pNETs associated with hypoxia signaling and metabolic reprogramming. More recent work by Kahan Yossef et al (17), investigating metabolomics and transcriptomics profile of pNETs in VHL vs non-VHL patients, showed a unique profile of pseudohypoxic NETs, with an abundance of adenosine and its metabolites in pseudohypoxic tumors. The penetrance of pNETs in *VHL* carriers ranges between 8% and 17% and tumors are typically nonfunctioning but may be malignant (18). The *VHL* locus is often inactivated in sporadic pNETs through nonmutational mechanisms and is associated with reduced survival rates (19). Sporadic and hereditary pNETs with *VHL* inactivation are both associated with upregulated HIF-2α activity and the expression of hypoxia-responsive genes. To date, molecular drivers of pNETs have not been linked to *EPAS1*.

The vast majority of genetic variants in *EPAS1* are somatic mosaic and/or occur in the oxygen-dependent degradation (ODD) domain (residues 529-540); however, variants 3′ of the ODD have been identified affecting residues 766, 785 to 789, and 834 (6, 20-23). Recent studies have suggested the role of environmental hypoxia exposure (eg, sickle cell disease, congenital cyanotic heart disease, and altitude) driving acquired somatic *EPAS1* variants in PPGLs and epigenetic modulation of *EPAS1* as an adaptation response; however, it is unclear whether they are inciting factors (24-27). In addition, it is uncertain whether chronic hypoxia increases the risk for other tumors. Thus far, none of the reported studies of patients with somatic mosaic PZS and those with environmental hypoxia exposure have reported pNETs, except for somatostatinoma(s) (28). There have been reports of a somatic *EPAS1* variant in a pNET (p.Pro531His) and germline *EPAS1* variant in an individual with concomitant VHL syndrome and an additional case of a pNET (with germline variants in *VHL* Trp117Ser and *EPAS1* p. His194Arg) (20, 29).

Belzutifan, a potent HIF-2α inhibitor (PT2977), was first evaluated in VHL-associated renal cell carcinoma and showed therapeutic benefit with objective response rates of 49% (30). It has likewise proven to be effective in other VHL disease cancer phenotypes, with a 91% therapeutic response rate in patients with pNETs (30, 31), suggesting a role of belzutifan in treating *EPAS1*-associated pNETs. Recent studies indicate that belzutifan has activity in all clear cell renal carcinoma with an objective response rate of 21.9% to 25% at a median follow-up of more than 25 months (32, 33). In addition, belzutifan has been used in cases of PZS with therapeutic effects on hemoglobin, erythropoietin, catecholamines, blood pressure control, and tumor control (34-36) along with advanced or metastatic PPGLs (37). Belzutifan has recently gained US Food and Drug Administration approval for usage in clear cell renal carcinoma as well as PPGL in the Unites States. Herein, we describe a novel case of pNET in the absence of synchronous PPGLs in an individual with PZS and investigate the phenotype of pNETs in a case series of patients with somatic mosaic pathogenic variants in *EPAS1*.

## Materials and methods

### Study design and patient selection

All patients included in the study provided written informed consent in accordance with the ethical standards established by the Declaration of Helsinki. The index patient was recruited to the Molecular Pathology of Human Genetic Disease Study (South Birmingham REC CA/125) at Addenbrooke's Hospital. Likewise, the institutional review board of the Eunice Kennedy Shriver National Institute of Child Health and Development (NICHD, ClinicalTrials.gov identifier: NCT00004847) approved the PPGL clinical study protocol. Patients with paraganglioma(s), erythrocytosis, and confirmed *EPAS1* somatic mosaic variant in the NICHD study met the criteria of PZS.

### Cohort evaluation and genetic analysis

The pathogenic *EPAS1* variant (Asp539His) in the index case was first identified on NHS clinical sequencing endocrine neoplasia and hereditary erythrocytosis panels by next-generation sequencing. Subsequently, the variant allele frequency both in the pNET and blood of the index patient were evaluated using custom-designed Sanger sequencing primers targeting the ODD of *EPAS1*. For PZS patients at the NIH, variants in the *EPAS1* ODD were evaluated in circulating leukocytes and/or resected tumors using whole-exome sequencing, droplet digital polymerase chain reaction, peptide nucleic acid sequencing, and were previously shown to be somatic mosaic in patient tissue (6, 9, 38). Phenotypic data along with laboratory results were collected from respective institutional electronic health records.

## Results

### Case presentation

A male infant was referred to the ophthalmology team with concerns regarding severe visual impairment and was registered as blind secondary to bilateral congenital retinal dysplasia at age 3 months. His family history was notable for factor V Leiden deficiency and a maternal uncle with seizures and intellectual disability of unknown etiology. Genetic testing for Norrie disease (associated with *NDP* pathogenic variants) and exudative vitreoretinopathy (linked to variants in *FZD4*, *LRP5*, and *TSPAN12*) were negative.

The patient underwent tonsillectomy at age 9 that was complicated by 2 episodes of postoperative hemorrhage. Blood tests showed a persistently elevated hemoglobin of 204 g/L (115-155 g/L), hematocrit of 0.677 (0.350-0.450 L/L), platelets of 134 × 10^9^/L (150-400 × 10^9^/L), and prolonged prothrombin time of 14 seconds (9.8-12.6 seconds). Blood film showed packed red cells with morphologically normal white cells and platelets, white cell differential was normal, and his blood tests were otherwise unremarkable except for a marginally elevated total bilirubin of 16 μmol/L (0-14 μmol/L).

**Figure 2:**
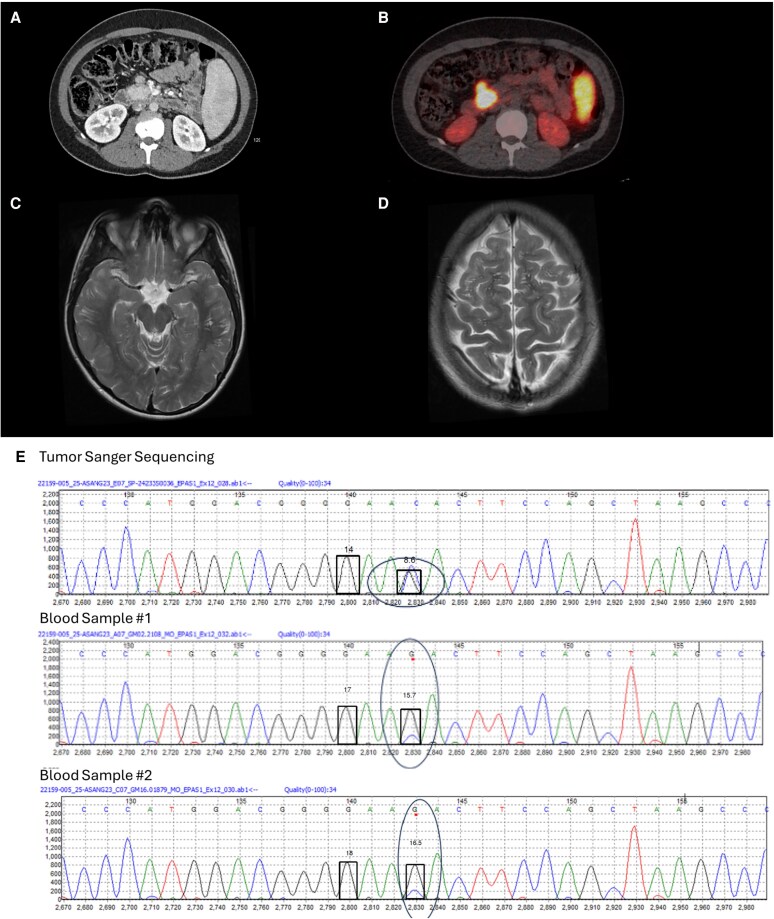
*HIF2A*-associated nonfunctional pancreatic neuroendocrine tumor. A, An axial contrast-enhanced computed tomography (CT) image of the abdomen and pelvis demonstrating a 30-mm pancreatic neuroendocrine tumor in the uncinate process and adjacent 10-mm lymph node. B, A sagittal fused image from a [^68^Ga]-DOTATATE positron emission tomography–CT illustrating avidity in the pancreatic mass and adjacent node. Axial T2-weighted imaging of the brain shows multiple visible and prominent perivascular spaces in C; the bilateral subcortical white matter of the temporal lobes; and D, the bilateral subcortical white matter of the frontal lobes. E, *EPAS1* variant allele frequency both in germline (sample 1, 15.2%; sample 2, 15.4%) and somatic tissue (pancreatic neuroendocrine tumor) (42.8%) in index case. Created in BioRender. Cole, Y. (2025) https://BioRender.com/z607ez6.

He was referred to the pediatric hematology team, who performed extensive investigations for primary and secondary erythrocytosis. Polymerase chain reaction testing of *JAK2* V617F and JAK2 exon 12 pathogenic variants were both negative, hemoglobin electrophoresis was normal, and clotting factor assays were normal, but his erythropoietin level was significantly elevated at 614 U/L (5-25 U/L) and iron studies were consistent with iron deficiency. The patient had serial whole-body magnetic resonance imaging (MRI), ultrasound, echocardiogram, and [^18^F]-FDG positron emission tomography–computed tomography (PET-CT) imaging over a period of 3 years that was negative for any cardiac congenital abnormality or erythropoietin-secreting source such as a hemangioblastoma, revealing only mild splenomegaly. Differential renal venous sampling was performed but was normal. MRI imaging of the head also demonstrated prominent perivascular spaces in the subcortical white matter of both cerebral hemispheres of unclear clinical significance and bilateral enophthalmos (Fig. 2). Bone marrow aspirate and trephine revealed a hypercellular marrow with erythroid hyperplasia and no features of dysplasia or increased blasts. The possibility of a pathogenic variant in the oxygen-sensing pathway or high-affinity hemoglobin was discussed, but genetic screens for *VHL*, *PHD2*, and *EPAS1* were initially negative.

In the interim, the patient was commenced on a program of intermittent venesection. Following a period of suboptimal hematocrit control, CT imaging was repeated for abdominal pain. This showed a new nonocclusive thrombus in the splenic vein in addition to known splenomegaly, features suggestive of noncirrhotic portal hypertension and a pancreatic lesion. He was started on oral anticoagulation, and endoscopic ultrasound was performed showing a 24-mm uncinate process lesion and an adjacent 10-mm lymph node. Fine-needle aspirate from both lesions was in keeping with a well-differentiated NET, showing cells with granular, coarse hyperchromatic chromatin patterns and scant eosinophilic cytoplasm that were positive on immunohistochemical staining for MNF116, synaptophysin, chromogranin A, and CD56. MIB-1 proliferation index was less than 3%. Clinically, the patient reported no symptoms/signs of hormone excess; specifically, he had no gastrointestinal (GI) symptoms and fasting gut peptide screening revealed a normal somatostatin level. Subsequent staging with a gallium-68 [^68^Ga]-DOTATATE PET-CT scan demonstrated high somatostatin receptor expression in the pancreatic lesion and adjacent node, with no other evidence of metastatic disease (see Fig. 2). The working diagnosis was that of a nonfunctioning pNET and after careful discussion, a Whipple resection was performed. Histology confirmed a grade 2, well-differentiated pNET, ENETS stage pT2 N1 R0.

A liver biopsy was arranged to investigate noncirrhotic portal hypertension and demonstrated features of portosinusoidal vascular disorder with an overall mild portal and perisinusoidal fibrosis. Esophago-gastroduodenoscopy was negative for any varices. In view of this new diagnosis of a pNET, genetic testing included an endocrine neoplasia panel (*AIP*, *CDC73*, *CDKN1B*, *MEN1*, *RET* exons 5, 8, 10, 11, and 13-16), and a repeat hereditary erythrocytosis panel (R405) by next-generation sequencing was also performed. These analyses were performed using the standardized genetic testing panels commissioned through the NHS. A mosaic likely pathogenic *EPAS1* variant c.1615G > C p. (Asp539His) was identified in germline blood samples taken at age 2 and 22, with a variant allele frequency of 13%.

Targeted Sanger sequencing also identified the same *EPAS1* variant in the pNET sample at a variant allele frequency of 42.8% (see Fig. 2). In addition, targeted Sanger sequencing validated the germline results: The variant allele frequency in the blood samples were 15.2% and 15.5%. Using American College of Medical Genetics and Genomics criteria for pathogenicity, the Asp539His variant met the criteria for likely pathogenicity with the following lines of evidence: PM1, PM2, PP3, PS3, and PS4. The variant was not identified in gnomAD nor ExAC (PM2) and is located in a hot-spot region of the oxygen dependent degradation domain of EPAS1 (PM1). Ferens et al reported the class 1 Asp539Tyr and Asp538Asn variants affect binding with PHD2, leading to increased transcriptional activity of EPAS1 (PS3) (39). In silico prediction models are congruent and predict the variant to be pathogenic (PP3): SIFT 0 (deleterious), Polyphen score 1 (probably damaging), CADD score of 31 (deleterious, top 0.1%), and an AlphaMissense score of 0.9977 (likely pathogenic). Somatic sequencing using a commercial gene panel assay via the TruSight Oncology 500 (TSO500) assay, which interrogates more than 500 cancer-related genes, was performed on DNA extracted from the pNET and no additional somatic variants were identified. Serial plasma metanephrines have been normal to date with no evidence of synchronous PPGLs on cross-sectional imaging or [^68^Ga]-DOTATATE PET-CT. Serial imaging has not identified recurrence of the pNET almost 2 years post surgery.

### Review of pancreatic phenotypes in mosaic EPAS1 (HIF2A) gain-of-function Pacak-Zhuang syndrome

**Figure 3:**
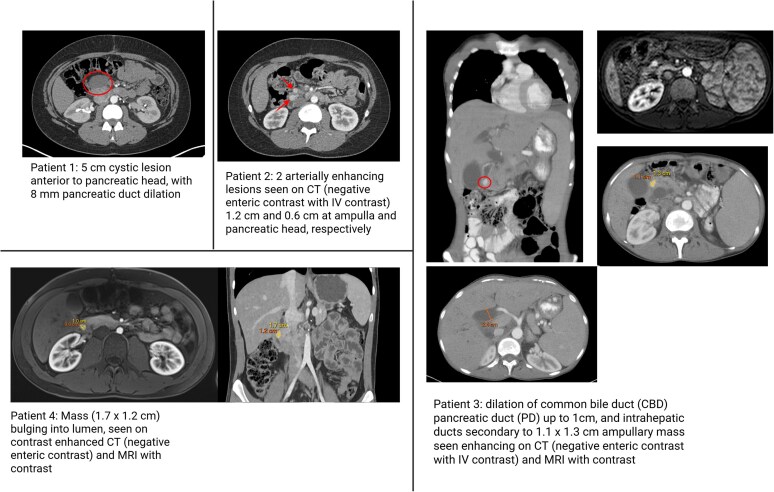
Somatostatinoma imaging in a Pacak-Zhuang syndrome cohort. Computed tomography (CT) images are provided for 4 individuals with somatostatinomas. Masses are denoted with either a circle or arrows along with measurements. Only a postsurgical contrast-enhanced CT imaging scan is available for patient 1 and illustrates a postsurgical cyst with pancreatic duct dilation. Patients 2 and 3 also demonstrate pancreatic duct dilation in addition to a somatostatinoma. Patient 5 underwent imaging and surgical resection at an outside institution prior to referral; although the imaging was not available for review, pathology confirmed the diagnosis of somatostatinoma. Created in BioRender. Cole, Y. (2025) https://BioRender.com/9qqp24l.

*EPAS1* somatic mosaic variants are a hallmark of NETs in PZS. In the 15 cases at the NIH, 5 individuals, followed for at least 11 years since first tumor evaluation, developed histologically confirmed pNET with oftentimes dilation of the pancreatic duct, in addition to PPGL(s) at or after the diagnosis of erythrocytosis (Fig. 3). Among those 5 patients, erythrocytosis was first diagnosed at a median age of 2 years (range, birth-7 years). The median age at diagnosis of PPGL was 24.4 years (range, 14-39 years), while pNETs were diagnosed at a median age of 29 years (range, 21-39 years) and metastatic pNETs occurred in 3 out of 5 cases. The cohort was predominantly female, with a female-to-male ratio of 4:1. The median somatostatin level was 47.6 pg/mL (normal range up to 25 pg/mL) across the group (range, 12-109 pg/mL), including a patient with normal somatostatin levels, similar to our reported index case, thus a normal somatostatin level does not exclude the diagnosis of NET in patients with PZS. Despite having a pathogenic variant at codon 539, similar to this case study, patient 1 in the NIH cohort had a functional somatostatin-producing pNET and had postsurgical cystic changes (see Fig. 3). All cases presented symptomatically with nonspecific generalized GI symptoms/signs and somatostatin secreting pNETs at an older onset (age range, 21-39 years) than this case study and with multiple tumors, local recurrence, and distant metastasis in 75%, 75%, and 50%, respectively (Table 1). Resected pNETs had *EPAS1* variants at a variant allele frequency of 31% to 56% of the tumor content, a similar percentage to our index case (42.8%). Critically, 2 of the individuals developed recurrence and metastasis and 3 individuals (patients 1, 2, and 4) were commenced on belzutifan after recurrence of PPGL and without surgical resection. To date, patient 2 has had limited disease progression on imaging after 17 months of treatment. Patients 1, 2, and 4 have had stable somatostatin levels since initiating belzutifan.

**Table 1:** Clinical phenotypes in Pacak-Zhuang syndrome

	PZS patient characteristics				
	Patient 1	Patient 2	Patient 3	Patient 4	Patient 5
Age at initial diagnosis, y	Erythrocytosis at age 2	Erythrocytosis at birth	Erythrocytosis at birth	Erythrocytosis at age 2	Erythrocytosis at age 7
Sex	F	F	F	M	F
PPGL, age at first diagnosis, y	39	18	14	15	36
PNET, somatostatinoma					
Age of initial diagnosis, y	39	23	29	21	36
Multiple somatostatinomas?	N	Y	Y	Y	Y
Somatostatin, <25 pg/mL	31	50	109	12	36
Age of recurrence, y	40	34 (possible recurrence with somatostatin elevated to 30)	32	NA	NA
Age of metastatic disease, y	39	NA	32	NA	36
Pathological grade (Ki67 index)	NA*^b^* (2%)	NA*^b^* (2%*^c^*)	NA*^b^*(NA*^b^*)	G1 (<3%), somatostatin positive on IHC, invading mucosa, submucosa, and muscularis propria	NA*^b^* (NA*^b^*), somatostatin positive on IHC, invading mucosa, submucosa, and muscularis propria
Catecholamines, plasma					
Norepinephrine, 80-498 pg/mL	1875	1760	10 951	257	775
Epinephrine, 4-83 pg/mL	24	7	100	<20	<20
Dopamine, 3-46 pg/mL	<25	20	28	<25	1091
Normetanephrine, 18-112 pg/mL	1993	858	4834	158	515
Metanephrine, 12-61 pg/mL	77	9	121	<12	<12
Variant analysis					
*EPAS1*	p.D539N	p.A530V	p.A530T	p.P531S	p.Y532C
Variant allele frequency, %					
Blood	Undetected	0.80%	12.70%	Undetected	Undetected
Nail	Undetected	Undetected	27.00%	NA	Undetected
Hair	Undetected	1.80%	12%	Undetected	1%
Buccal	3.80%	1.30%	17.80%	NA	Undetected
Tumor	NA*^a^*	31% and 37% (2 pancreatic lesions)	56%	40%	NA
GI symptoms	Early satiety, occasional nausea	Episodes of dull epigastric pain without clear triggers or relieving factors	Obstructive jaundice, chronic abdominal pain and nausea/vomiting	None; identified incidentally	Obstructive jaundice
Other phenotypes	EPO-dependent erythrocytosis, optic disc fibrosis, peripapillary gliosis and optic disc drusen in left eye, strabismus with amblyopia and esotropia of left eye, pancreatic cysts, liver lesion on MRI	EPO-dependent erythrocytosis, optic disc fibrosis, vascular tortuosity with dilated veins, peripapillary gliosis bilateral eyes, abnormal retinal vasculature with tortuous thickened veins and peripheral retinal pigment epithelial changes, bilateral renal cysts, breast cysts	EPO-dependent erythrocytosis, optic disc fibrosis, peripapillary gliosis and peripheral retinal vascular anomalies of both eyes, exotropia of left eye, hemangioblastoma-like lesion in left eye, marfanoid habitus, ascending aortic aneurysm	EPO-dependent erythrocytosis, optic disc fibrosis, optic nerve gliosis, macular edema and tortuous vessels primarily in right eye	EPO-dependent erythrocytosis, mild optic nerve gliosis, bilateral cataract (posterior subcapsular), optic disc fibrosis, HNPGLs, lung and cervical cysts

Clinical phenotypes and laboratory investigations are provided for 4 individuals with PNETs (somatostatinomas) from a cohort of PZS patients at the National Institutes of Health. Other phenotypes have been previously published (6). Age of onset is provided for each phenotype. For somatostatinomas, information regarding disease recurrence is provided.

Abbreviations: EPO, erythropoietin; F, female; GI, gastrointestinal; HNPGL, head and neck paraganglioma; IHC, immunohistochemistry; M, male; MRI, magnetic resonance imaging; N, no; NA, not available; PNET, pancreatic neuroendocrine tumor; PPGL, pheochromocytoma/paraganglioma; PZS, Pacak-Zhuang syndrome; Y, yes.

^*a*^Denotes that a variant allele frequency was not available on the tumor.

^*b*^Denotes information was not available/reported in histologic report.

^*c*^Ki67 index from one of the tumors resected.

## Discussion

This case study of a nonfunctioning NET in an individual with congenital erythrocytosis, bilateral cataracts, and splenomegaly is the first reported case of an individual with a nonfunctioning pNET and pathogenic *EPAS1* variant. This case study describes a new genetic etiology of pNETs. Approximately 10% of individuals with VHL disease, with higher frequencies in those with PPGL predisposing to missense variants, develop pNETs, which are often nonfunctioning and multifocal (18, 40, 41). Recently, proteomic assessment and differential gene expression of pNETs have identified the molecular subtypes metastasis-like tumors, associated with a poor prognosis, and are characterized by upregulated hypoxia signaling such as HIF-1α expression (42); however, underlying genetic pathogenic variants in hypoxia signaling pathways beyond *VHL* were not identified. The identification of direct binding of HIF-2α with β-catenin (43) supports the involvement of downstream HIF-2α signaling in the pathophysiology of pNETs. Landmark studies of the therapeutic efficacy with the potent small-molecule inhibitor of HIF-2α belzutifan in VHL-associated pancreatic lesions in patients with clear cell renal carcinoma (31) suggests that the hypoxia subtype of pNETs including *EPAS1* may also be amenable to the same therapy, and therefore assessment of the frequency of *EPAS1* variants in large cohorts of pNETs is warranted. However, it should be noted that, as seen with the initial testing in our case, mosaic *EPAS1* variants may not be detected by routine genetic testing and specific molecular investigations may be indicated if low-level mosaic variants are suspected. The efficacy of the HIF-2α inhibitor belzutifan has expanded from VHL-associated renal tumors to a role in all clear cell renal carcinomas and metastatic PPGL (33) and conceptualizes the larger translational implications of studying rare genetic syndromes and genotype-phenotype correlations. This case series illustrates an expansion of the current understanding of gene-disease phenotypes associated with *EPAS1,* including the association with functioning and nonfunctioning pNETs. The observation of portosinusoidal vascular liver disease, enophthalmos, prominent perivascular spaces within the brain white matter, and a nonfunctioning pNET in one individual in this case series supports the possibility of a spatiotemporal effect of the *EPAS1* mosaic variant and warrants further evaluation in a transgenic *EPAS1* murine model.

## Data Availability

All clinical data were obtained from electronic medical records at Cambridge University Hospitals and the NIH. The authors confirm that the data supporting the findings are available within the article. Data sharing is not applicable to this article as no datasets were generated or analyzed during the current study.

## Abbreviations

GI: gastrointestinal
MEN1: multiple endocrine neoplasia type 1
MRI: magnetic resonance imaging
*NF1*: neurofibromatosis gene
NICHD: National Institute of Child Health and Development
NIH: National Institutes of Health
ODD: oxygen-dependent degradation
PET-CT: positron emission tomography–computed tomography
pNET: pancreatic neuroendocrine tumor
PPGL: pheochromocytoma/paraganglioma
PZS: Pacak-Zhuang syndrome
*VHL*: von Hippel-Lindau gene
